# Fabrication of Cotton Linter-Based Adsorbents by Radiation Grafting Polymerization for Humic Acid Removal from Aqueous Solution

**DOI:** 10.3390/polym11060962

**Published:** 2019-06-02

**Authors:** Jifu Du, Zhen Dong, Yuxuan Pi, Xin Yang, Long Zhao

**Affiliations:** 1 School of Nuclear Technology and Chemistry & Biology, Hubei University of Science and Technology, Xianning 437100, China; duzidedu@163.com (J.D.); yuxuanpi@foxmail.com (Y.P.); sophieyangyifan@163.com (X.Y.); 2 State Key Laboratory of Advanced Electromagnetic Engineering and Technology, School of Electrical and Electronic Engineering, Huazhong University of Science and Technology, Wuhan 430074, China; zhendong@hust.edu.cn

**Keywords:** cotton linter, humic acid, adsorption, radiation grafting

## Abstract

Two kinds of cotton linter-based adsorbents were synthesized by grafting dimethylaminoethyl methacrylate (DMAEMA) on cotton linter via radiation grafting polymerization, followed by further quaternization (QCL) or protonation (PCL). The effect of radiation dose and monomer concentration on grafting yield was optimized. The synthesized adsorbents were characterized by Fourier transform infrared spectrometry (FT-IR), Thermogravimetric Analysis (TGA), scanning electron microscope (SEM) and X-ray photoelectron spectroscopy (XPS). The adsorption behaviors of the two adsorbents toward humic acid (HA) were investigated and discussed. pH effect studies showed that QCL was pH-independent, whereas PCL was just suitable for HA adsorption with pH < 6. The adsorption kinetics of the PCL and QCL adsorbent for HA removal were better described by pseudo-second-order kinetic mode and reached equilibrium in 40 min. The adsorption isotherms of the PCL and QCL adsorbent were well fitted with both Langmuir and Freundlich isotherm models, for which adsorption capacity reached 250 mg/g and 333 mg/g at pH 6, respectively. XPS analysis revealed the ratio of two amino group species at different pH, suggesting that the interaction mechanism of the adsorbent and HA was electrostatic adsorption.

## 1. Introduction

Humic acid (HA) is one of the major components of humic substances and it contains both hydrophilic and hydrophobic moieties, such as carboxylic, phenolic, carbonyl, and hydroxyl groups [1]. HA is a complex organic colloid and a relatively stable organic compound, therefore it is difficult to biodegrade under natural conditions. The presence of HA in natural waters would cause various environmental and health problems, such as undesirable color and taste of water [2], so efficient removal of HA in water treatment has attracted many environment and health considerations.

Membrane filtration, flocculation, oxidation, and biological methods have been investigated and applied to remove HA from water. But there are some problems with the practical application of those processes. For example, HA can easily result in membrane fouling when membrane filtration is used, the inorganic or organic flocculants have certain health hazards, the oxidation method is complex and has high operating costs, and biological methods need a long time and are less effective [3]. Comparatively, adsorption is considered one of the most effective water treatment techniques, as it is cost-effective, straightforward, and efficient in removing HA. Many adsorbents, such as bentonite [2], modified bentonite [1], montmorillonite [4], layer structured graphite oxide [5], and cross-linked carboxymethyl chitosan [6] have been investigated for HA removal, and the results showed that they are suitable for HA removal at a lower pH [6]. From the view point of practice, research should focus on how to broaden the pH range for HA removal.

The performance of the adsorption process depends on the type of adsorbent used. Adsorbents derived from natural polymers are desirable from the viewpoint of environment-conscious technologies. Cellulose, one of the most popular natural polymers, has received great attention as an adsorbent matrix, in terms of its hydrophilicity, biocompatibility, and abundance in nature. Adsorbent materials derived from cellulose have been widely studied [7]. Cotton linter, the relatively short fuzz left on cotton seed after the cotton ginning process, is the purest cellulose in chemistry, with hundreds of end uses. As an essentially regenerative natural resource and the by-product from cotton textiles, it has been applied as the substrate owing to its low cost and high cellulose content. In order to increase the adsorption capacity and adsorption rate, radiation treatments have been widely used to treat various lignocellulosic biomasses, including natural fibers, with the aim of improving their accessibility to solvents and reagents for their subsequent chemical modification and processing [8]. Radiation-induced graft polymerization (RIGP) is a convenient and powerful technique to introduce desirable functional groups on polymer surfaces for adsorption application [9,10]. Adsorbents prepared by radiation grafting have high adsorption velocity because the functional groups are mainly concentrated on the surface of polymer substrates [11]. In this study, quaternized cotton linter (QCL) and protonated cotton linter (PCL) were prepared by RIGP, following modification for HA removal from an aqueous solution. The effect of pH, contact time, and HA concentration on HA removal was investigated, and the adsorption mechanisms were studied by XPS.

## 2. Experimental

### 2.1. Materials

Cotton linter was obtained from Jinhanjiang refined cotton co., Ltd. (Jingmen, China). Dimethylaminoethyl methacrylate (DMAEMA) and Dimethylformamide (DMF) were supplied by Aladdin Chemical Co., Ltd. (Shanghai, China). 1-Bromohexane and was supplied by Macklin reagent Co., Ltd. (Shanghai, China). HCl, NaOH, and Hydroquinone were supplied by Sinopharm chemical reagent ltd. (Shanghai, China). The sodium salt of HA were purchased from Sigma-Aldrich (Darmstadt, Germany). All chemicals were used as received without further purification.

### 2.2. Preparation of Cotton Linter-Based Adsorbents

**Figure 1:**
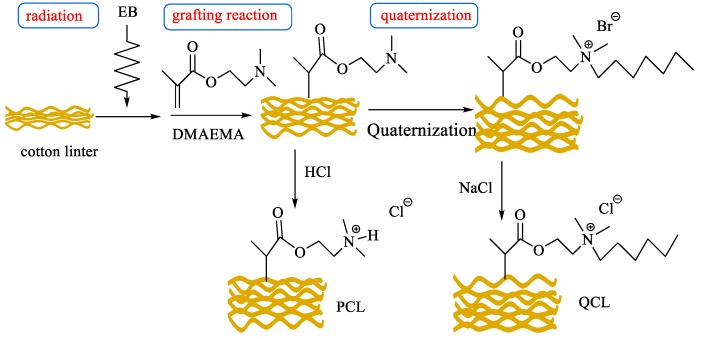
Procedure for the preparation of cotton linter-based adsorbents.

The cotton linter based adsorbents were prepared by electron beam (EB) pre-irradiation grafting polymerization; the procedure is illustrated in Figure 1.

A total of 2 g of dry cotton linter fibers with a diameter of 15 um were sealed in a polyethylene (PE) bag, which was then vacuum pumped. The DMAEMA solution with a concentration of 30 wt% was deoxygenated by bubbling nitrogen for 60 min. Hydroquinone with 200 mg/L was used as polymerization inhibitor. Then, 30 mL of the DMAEMA solution was injected into the PE bag. The bag was irradiated at a dose of 10–60 kGy (dose rate: 10 kGy/pass) by an EB accelerator (Wasik Associates INC, USA) at 1 MeV. The irradiated cotton linters were put into the 100 mL ethanol aqueous and acetone solution for 24 h, successively. Then, the sample was washed with deioned water, and dried in a vacuum at 50 °C. The grafting yield (GY) was calculated using Equation (1):

(1) $$\mathrm{GY}=\frac{W_{g}-W_{0}}{W_{0}}\times 100\%,$$

where W_0_ and Wg are the weights of the cotton linters before and after grafting, respectively.

Finally, some of the grafted samples were quaternized for 24 h in a 50% 1-bromohexane and 50% DMF solution at 70 °C, then immersed in 1 M NaCl solution to substitute the Br^−^ ions with Cl^−^, −10^−7^ thus QCL was obtained. Some of the grafted samples were protonated by 1 M HCl for 24 h, the resultant was PCL. Finally, the modified cotton linters were washed and dried to a constant weight.

### 2.3. Characterization

A Netzsch TG290F3 (Selb, Germany) was used for the Thermogravimetric Analysis (TGA) analysis at the temperature from 0 °C to 800 °C. The Fourier transform infrared spectroscopy (FTIR) spectra were recorded in the transmittance mode on an FTIR-650 spectrometer (Thermo, Waltham, MA, USA) in the range of 400–4000 cm^−1^.

Morphologies of the sample were observed by a Tescan Vega3 scanning electron microscope (SEM) (Brno, Czech) at the accelerate voltage 10 kV.

The X-ray photoelectron spectroscopy (XPS) analysis was performed with an AXIS-Ultra instrument (Kratos Analytical, German), using monochromatic Al Ka radiation and low energy electron flooding for charge compensation. The data were converted to VAMAS file format and imported into Casa XPS software package for manipulation and curve fitting.

The pH of the point of zero charge (pH_pzc_) was tested by the pH drift method. A total of 10 mL 0.01 M NaCl solution were adjusted to different pH values between 2 and 12 at 298 K by 0.1 M HCl or NaOH, and the QCL or PCL (0.10 g) were added into the solution. After 24 h, the final pH of the solution was measured. The pH point where pH (final) = pH (initial) was taken as the pH_pzc_ of QCL and PCL.

### 2.4. Analysis of HA Concentration in Test Solution

The concentration of HA was determined at 440 nm using a UV-vis spectrophotometer (UV-3600, SHIMADZU, Kyoto, Japan). The correlation coefficient R^2^ of the standard curve was 0.9997.

### 2.5. Batch Experiments

A total of 0.05 g of adsorbent was dispersed in a 50 mL breaker containing the HA aqueous solution. The beaker was shaken in a shaker at 30 °C. The amount (Q_t_) of HA adsorbed onto the adsorbents was calculated by Equation (2):

(2) $$Q_{t}=\frac{{(C}_{0}{-C}_{t})\times V}{m},$$

where C_0_ and C_t_ were the initial and final total concentrations of HA, respectively; V was the volume of the ion solution, and m was the weight of dry adsorbent.

Three parallel experiments were performed, and the average of the results was used as the adsorption result, with the errors within 2%. pH was adjusted with 0.1 mol/L HCl or 0.1 mol/L NaOH.

Desorption studies were conducted by batch experiments. The 0.05 g HA loaded adsorbent (after HA adsorption at the initial pH of 6.0 with a 0.05 g PCL or QCL in 50 mL of 20 mg/L HA solution for 24 h) was placed into 50 mL of HCl or NaOH solution for 24 h, the suspension was filtered, and the HA concentration was determined in the supernatant. The desorption efficiency was calculated by the ratio of the amount of HA desorbed in the elution medium and the amount of HA adsorbed on PCL or QCL.

## 3. Results and Discussion

### 3.1. Preparation

**Figure 2:**
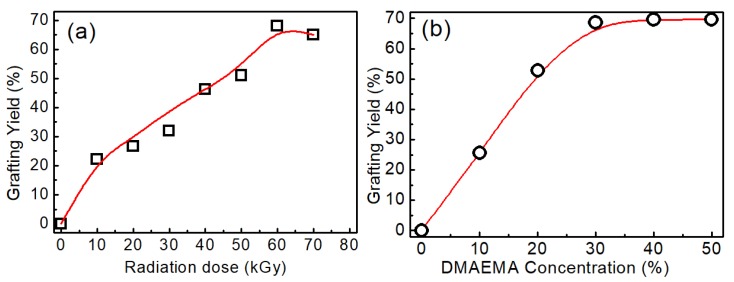
Effect of radiation dose (**a**) and dimethylaminoethyl methacrylate (DMAEMA) concentration (**b**) on grafting yield.

The effect of radiation dose on grafting yield (GY) was investigated. Figure 2a shows the influence of the radiation dose on GY at a DMAEMA concentration of 30 wt%. The GY firstly increased with radiation dose up to 68.8% at 60 kGy, and then decreased slightly. It is widely accepted that radiation graft polymerization is mainly a free radical polymerization mechanism, and the grafting polymerization is controlled by the total amount of free radicals formed in both substrates and the monomer solution. Thus, a higher dose means a larger amount of free radicals, and more monomers will graft on the substrate. In the case of co-irradiation grafting polymerization, higher radiation dose also causes the formation of homopolymer P(DMAEMA). Hence, 60 kGy was selected as the optimum condition for the preparation of DMAEMA grafted cotton linter.

The influence of DMAEMA concentration on GY was investigated with a radiation dose of 60 kGy, and the results are shown in Figure 2b. The GY increased with the DMAEMA concentration, and was up to the maximum at 30 wt%. In this study, the grafted samples with GY 68.8% at the dose of 60 kGy and 30% DMAEMA concentration were chosen for further protonation or quaternization.

### 3.2. Characterization

#### 3.2.1. FTIR Analysis

**Figure 3:**
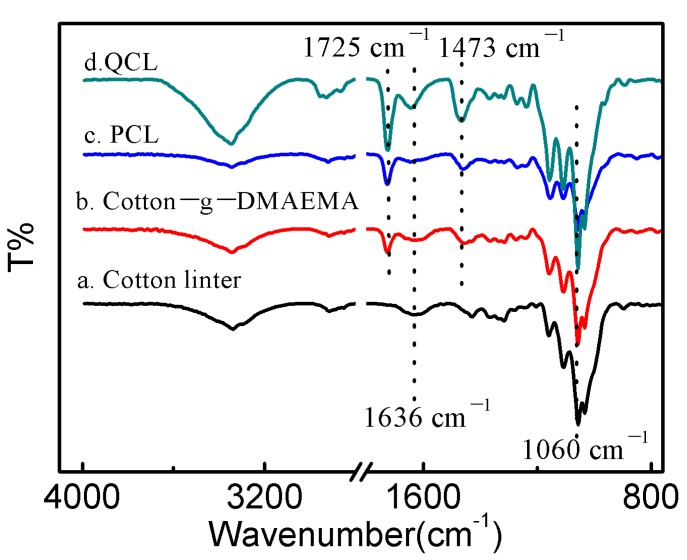
Fourier transform infrared spectrometry spectra of cotton linter (**a**), cotton-g-DMAEMA (**b**), PCL (**c**) and QCL (**d**) adsorbent.

Figure 3 shows the FTIR spectra of the original cotton linter (a), cotton-g-DMAEMA (b), PCL (c), and QCL (d). In Figure 3a, the typical absorption bands of cellulose are observed, the band at 3350 cm^−1^ was due to O–H stretching vibration. The band at 2918 cm^−1^ corresponds to the C–H stretching vibration. The adsorption band around 1636 cm^−1^ was attributed to H–O–H bonding. The band at 1060 cm^−1^ was assigned to C–O stretching vibration for cellulose [7,12].

New peaks appeared after DMAEMA was grafted, as shown in Figure 3b, the adsorption bands at 1725 cm^−1^ were ascribed to the stretching vibration of the carboxyl group of DMAEMA [13,14]. The results show that DMAEMA was successfully grafted onto cotton linter. There was no new obvious adsorption band of PCL that appeared. A new band of QCL at 1473 cm^−1^ in Figure 3d was the characteristic band of the quaternary ammonium group [14], which showed that a quaternization reaction occurred.

#### 3.2.2. Thermal Analysis

**Figure 4:**
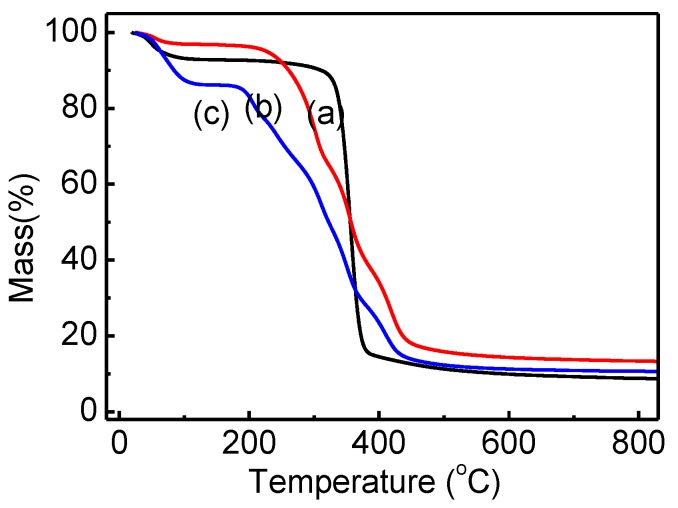
TG analysis of cotton linter (**a**), cotton-g-DMAEMA (**b**) and QCL (**c**).

Figure 4 shows the TG analysis of cotton linter (a), cotton-g-DMAEMA (b) and QCL (c). The first weight loss region lower than 100 °C was due to a loss of water. It can be noted that the thermal degradation behavior of cotton linter involved a one-stage process. The main weight loss occurred between 350 °C and 380 °C, suggesting that cotton linter has good thermal stability. The TG curves of the cotton-g-DMAEMA and QCL samples showed lower decomposition temperature, the main weight loss region was between 220 °C and 450 °C for PCL and 180 °C and 420 °C for the QCL.

#### 3.2.3. SEM Morphologies

**Figure 5:**
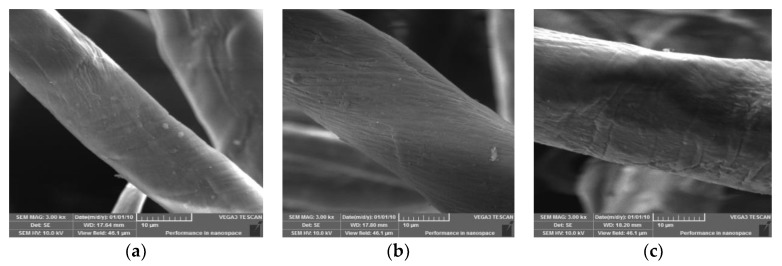
SEM photographs of cotton linter (**a**), cotton-g-DMAEMA (**b**) and QCL samples (**c**).

SEM photographs of the cotton linter, cotton-g-DMAEMA, and QCL samples were examined at a magnification of 3000× using SEM shown in Figure 5. The change in fiber morphology after grafting and chemical modifications were clear in the SEM morphologies. The diameter of the cotton-g-DMAEMA and QCL were become larger, and the surface of QCL was quite rough compared with the initial cotton linter, with wrinkles and grooves. The roughness of the chemically modified samples was helpful for absorption.

### 3.3. Batch Experiments

#### 3.3.1. Effect of pH and Point of Zero Charge

**Figure 6:**
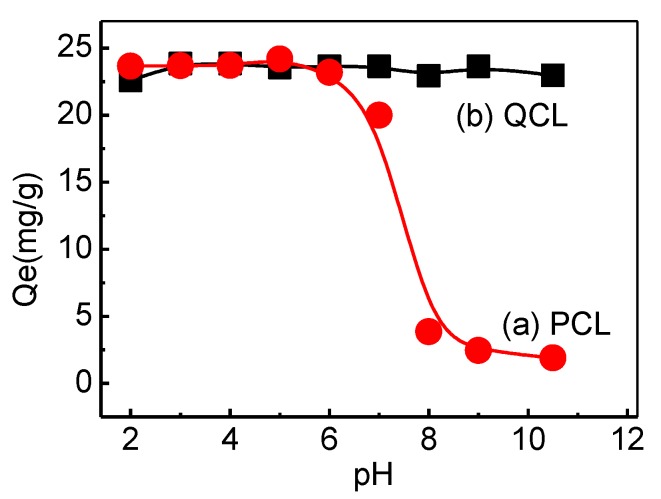
Effect of pH on HA adsorption on QCL (**a**) and PCL (**b**) adsorbents (C_0_ = 25 mg/L; V = 50 mL; adsorbent mass = 0.05 g; contact time 24 h).

HA contains both hydrophobic and hydrophilic moieties, as well as many chemically functional groups. The negative charge of HA molecules in aqueous solution is a result of the existence of carboxylic and phenolic groups in its structure and their deprotonation. The pH of the solution appears to be very important in relation to the adsorption result, due to how pH changed the molecular structure of the HA and adsorbent. The pH of the point of zero charge (pH_pzc_), tested by the pH drift method, was 6.5 and 11 for PCL and QCL, respectively. These results show that PCL was positive for pH < 6.5 and QCL was positive for pH < 11, due to the protonation features of amine groups. Figure 6 showed the HA adsorption capacity of PCL and QCL adsorbents with different pH levels. In curve (a) of the PCL adsorbent, the adsorption capacity of HA was found to be unchanged with an increasing initial pH < 6, and then a sharp decrease in HA adsorption was observed at a pH between 6 and 8. It was found that there was hardly any adsorption at pH > 10. Since PCL becomes negative at pH > 6.5, the deprotonation of the PCL adsorbent leads to a decrease in adsorption performance. In curve (b) of the QCL adsorbent, the absorption capacity maintained a high level at all pH ranges, which shows that the QCL adsorption process was little affected by different pH levels. According to these results, the initial pH of 6, without adjustment, was chosen as the optimum value for further investigation.

#### 3.3.2. Adsorption Kinetics

**Figure 7:**
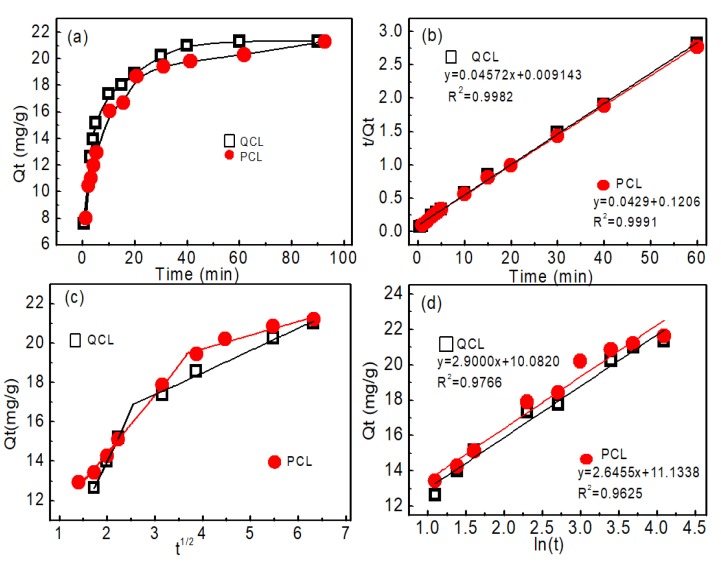
Adsorption kinetics of HA removal onto QCL and PCL. (**a**) Effect of adsorption time, (**b**) pseudo-second-order model, (**c**) intra-particle diffusion model, and (**d**) Elovich kinetic model (V = 50 mL, C_0_ = 25 mg/L, adsorbent mass = 0.05 g, pH: 6).

The adsorption equilibrium time is of great importance for studying the affinity of the adsorbent to HA. As can be seen from Figure 7, the absorption capacity of HA rapidly increased within the first 20 min for both the QCL and PCL adsorbents, and then increased gradually with time until it reached equilibrium at about 40 min.

Usually, the mechanisms of the adsorption processes were investigated through both pseudo-first-order and pseudo-second-order kinetic equations, as expressed by Equations (3) and (4), respectively. The initial adsorption rate h_0_ (mg/g/min) (t→0) can be calculated by Equation (5) [15].

(3) $$\ln(q_{e}-q_{t})=\ln q_{e}-kt,$$

(4) $$\frac{t}{q_{t}}=\frac{1}{k_{2}q_{e}^{2}}+\frac{t}{q_{e}},$$

(5) $$h_{0}=k_{2}Q_{e}^{2}\;,$$

where *q_e_* (mg/g) and *q_t_* (mg/g) were the amounts of HA adsorbed per gram of adsorbent at the equilibrium and at time t (h), respectively. *k*_1_ and *k*_2_ refer to the rate constant for the pseudo-first-order and the pseudo-second-order models, respectively.

The parameters *q_e_*, *k*_1_, and *k*_2_ of QCL and PCL adsorbents are listed in Table 1. For QCL adsorbents, the correlation coefficients (R^2^) for the pseudo-first-order and the pseudo-second-order kinetic models are 0.7772 and 0.9982, respectively. For PCL adsorbents, the correlation coefficients (R^2^) for the pseudo-first-order and the pseudo-second-order kinetic models are 0.7207 and 0.9991, respectively. The plots of both adsorbents obeyed the pseudo-second-order model well, as shown by the higher correlation coefficient, suggesting there was a chemical adsorption process. Besides, the rate constant k_2_ and initial adsorption rate h_0_ (10.95 mg/g/min) of QCL adsorbent was much larger than that of PCL adsorbent (8.309 mg/g/min). Therefore, QCL adsorbent had higher adsorption efficiency than PCL adsorbent.

The kinetics results can be analyzed by the intra-particle diffusion model proposed by Weber and Morris. This model is commonly used to study the transportation of the dissolved adsorbate from the solution to the used adsorbent [16]. The linear form of the intra-particle diffusion kinetic equation can be expressed as in Equation (6):

(6) $$q_{t}=k_{p}t^{1/2}+C,$$

where the *K_p_* parameter is the reaction rate constant (mg/g·min^1/2^) and the C parameter is the intercept which can be identified based on the thickness of the formed boundary layer. The fitting results with the model were represented graphically through the non-linear plotting of *q_t_*, versus t^0.5^ (Figure 7c), and the key parameters are listed in Table 1. The resulting curves of QCL and PCL for HA showed several adsorption steps without passing through the origin. Thus, the removal processes of the HA contained more than one adsorption mechanism, i.e., the system involved intra-particle diffusion of HA into QCL and PCL and other adsorption processes.

The Elovich kinetic model is commonly used to describe the second-order kinetics, if the adsorbent surfaces are energetically heterogeneous, and the kinetic models of chemical adsorption mechanisms [17]. The linear form of the Elovich equation is expressed in Equation (7):

(7) $$q_{t}=\frac{1}{\beta }\ln(\alpha \beta )+\frac{1}{\beta }\ln(t),$$

where α is the initial adsorption rate (mg/min) at contact time *t* = 0 min, and *β* is the extent of the surface coverage and activated energy (g/mg).

The Elovich model constants, *α* and *β*, were calculated from the linear regression plotting of *q_t_* versus ln (*t*), and listed in Table 1. The adsorption of HA showed medium fitting with good correlation coefficients on QCL (R^2^ = 0.9766) and on PCL (R^2^ = 0.9625), which indicates the energetically heterogeneous nature of QCL and PCL adsorbents for the adsorption of HA.

#### 3.3.3. Adsorption Isotherms

**Figure 8:**
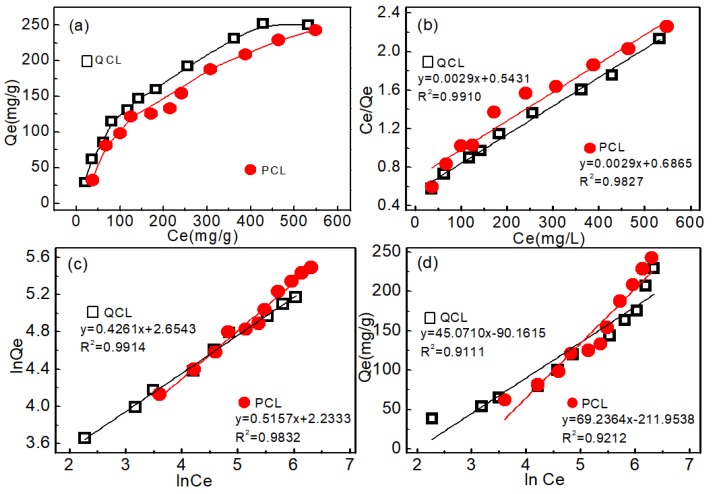
Adsorption isotherms: (**a**) effect of HA concentration, (**b**) Langmuir isotherm model, (**c**) Freundlich isotherm model, and (**d**) Temkin isotherm model (initial concentration: 50–800 mg/L, temperature 30 °C, pH 6, contact time: 24 h).

The equilibrium modeling and isotherm studies of the adsorption systems were used widely to investigate the adsorption mechanism and the expected distribution of the adsorbate between water and the adsorbent. Figure 8 shows the experimental isotherm data of HA adsorption onto QCL and PCL, conducted at 30 °C and pH 6 with a HA concentration range of 50–800 mg/L.

The Langmuir isotherm model was applicable to adsorption on homogeneous surfaces. The Langmuir isotherm equation can be expressed by Equation (8):

(8) $$\frac{\mathrm{Ce}}{q_{e}}=\frac{Ce}{q_{m}}+\frac{1}{K_{L}q_{m}},$$

where *C_e_* represents the equilibrium concentration of HA (mg/L), *q_e_* is the equilibrium adsorbed amount of HA per gram adsorbent (mg/g), *q_m_* refers to the theoretical maximum adsorption capacity (mg/g), and *K_L_* is the binding constant related to the adsorption energy (L/g). *q_m_* and *K_L_* are calculated from the slope and the intercept of the linear line of *C_e_*/*q_e_* versus *C_e_*, respectively.

The linear fitting of the data with the model is shown in Figure 8b. The adsorption results were highly fitted with the model, which was reflected in the values of the estimated determination coefficient. Hence, the adsorption of HA on QCL and PCL were monolayer adsorptions. The model parameters were calculated and are presented in Table 2. The obtained maximum adsorption quantities (Q_max_) of HA were 250 mg/g, and 333.3 mg/g for QCL and PCL, respectively. This was relatively higher than other adsorbents shown in Table 3.

The Freundlich isotherm model is commonly applied to describe adsorption for heterogeneous surfaces, according to Equation (9):

(9) $$\ln q_{e}=\ln K_{F}+\frac{1}{n}\ln C_{e},$$

where, *K_F_* is the Freundlich constant related to adsorption amounts of adsorbent (mmol/g), and n is the Freundlich exponent related to adsorption intensity [18]. The parameters of the Freundlich isotherm model were calculated from the linear regression plotting of ln *q_e_* versus ln *C_e_* and recorded in Table 2. The adsorption of HA on QCL and PCL were well fitted with this model, with a high correlation coefficient, which means that the adsorption may be heterogeneous in nature and occurs in a multiple layer form. In general, *n* > 1 illustrates that the adsorbate is favorably adsorbed on the adsorbent. The value of *n* was greater than unity, indicating that HA was favorably adsorbed by both of the two adsorbents [17,19].

The Temkin isotherm assumes that the heat of adsorption decreases linearly with coverage of the adsorbate and adsorbent interactions, and adsorption is characterized by a uniform distribution of binding energy, up to some maximum binding energy. The Temkin isotherm is represented by the linear equation in Equation (10):

(10) $$q_{e}=B_{T}\ln K_{T}+B_{T}\ln C_{e},$$

where *K_T_* is the equilibrium binding constant corresponding to the maximum binding energy and constant, and *B_T_* is the heat of adsorption corresponding to the intensity of adsorption. *K_T_* and *B_T_* were determined from a plot *q_e_* versus ln *C_e_*, and the corresponding parameters along with correlation coefficients are listed in Table 2.

#### 3.3.4. Desorption Experiments

It was practical to regenerate the adsorbent in order to make the adsorption process more economical. Desorption experiments of PCL and QCL were performed using four kinds of elution reagents, and the desorption efficiencies are shown in Table 4. It was found that the desorption efficiency increased with the increase in the concentration of NaOH. The highest desorption efficiency (75.98%) for PCL was given by a 3 M NaOH solution. The desorption efficiencies of HA from QCL with HCl and NaOH were similar and below 40%. These results indicate that the adsorption and desorption of HA onto PCL and QCL were probably through ion-exchange. To improve the desorption efficiency of HA from PCL and QCL, conditions of elution need to be further studied and optimized.

### 3.4. XPS Analysis

**Figure 9:**
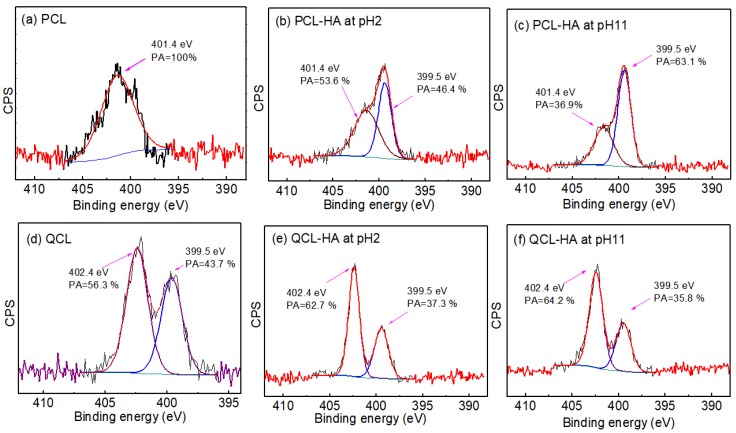
The high resolution X-ray photoelectron spectroscopy (XPS) N1s spectra of PCL, QCL before and after HA adsorption at different pH: (**a**) PCL, (**b**) PCL after HA adsorption at pH 2, (**c**) PCL after HA adsorption at pH 11, (**d**) QCL, (**e**) QCL after HA adsorption at pH 2, (**f**) QCL after HA adsorption at pH 11.

XPS analysis is a powerful tool to verify the adsorption mechanism. The high-resolution XPS N1s spectra of PCL and QCL adsorbents before and after adsorption of HA at different pH levels were performed, and their curve fitting analyses are shown in Figure 9. The N1s spectra of PCL could be curve-fitted with only one peak component at a Binding energy (BE) of 401.4 eV, due to the protonated amino group (–NH^+^(CH_3_)_2_), which showed the PCL adsorbent was completed protonated in 1M HCl. After HA adsorption at pH 2 and 11, the peaks of N1s were at a BE of 399.5 eV, owing to the –N(CH_2_)_3_ group which appeared, meaning that the PCL was partly deprotonated. Calculated from the peak area (PA), the ratio of the –NH^+^(CH_3_)_2_ changed from 53.6% to 36.9% when the pH value changed from 2 to 11, suggesting that the deprotonation of the amino group increased with the increase in pH value. On the other hand, the N1s spectra of the QCL could be curve-fitted with two peaks at BEs of 399.5 eV and 402.4 eV, owing to –N(CH_3_)_2_ and –N(CH_3_)_3_^+^ groups, respectively [27]. The ratio of the –N(CH_3_)_3_^+^ group and the –N(CH_3_)_2_ group was 56.3:43.7, which shows that the –N^+^(CH_3_)_3_ groups were incompletely quaternized. The ratio of the two –N(CH_3_)_3_^+^ groups and the –N(CH_3_)_2_ group was changed slightly due to HA adsorption. The ratio of the two species was almost the same at pH 2 and 11, which shows that the amount of the –N(CH_3_)_3_^+^ group on QCL was not changed with varying pH. The change trend was consistent with pH effect studies. Based on those results and pH effect studies, it is considered that the adsorption mechanisms of HA onto PCL and QCL were electrostatic adsorptions between negative HA and positive functional groups (–NH^+^ (CH_3_)_2_ and –N^+^(CH_3_)_3_) on adsorbents.

## 4. Conclusions

Two cotton linter-based adsorbents (PCL and QCL) were synthesized for HA removal by grafting DMAEMA onto cotton linters, using radiation grafting and further chemical modification. The adsorption performance of the two adsorbents to HA were discussed. pH studies and XPS analysis showed that PCL can only be used at pH < 6 and QCL can be used without the effect of pH values. The adsorption kinetics of both adsorbents for HA removal presented that adsorption kinetics were well described by the pseudo-second-order kinetic model. The adsorption isothermal of PCL and QCL were well fitted with both Langmuir and Freundlich isotherm models, whose adsorption capacity for HA reached 250 mg/g and 333.33 mg/g, respectively. XPS analysis revealed the interaction mechanism of adsorption of HA by PCL and QCL was electrostatic adsorption. In other words, the PCL adsorbent had higher adsorption capacity and QCL had the property of being pH-independent for HA removal. This work may provide new candidates of adsorbents for HA removal and a new renewable approach of natural biomasses.

## Figures and Tables

**Table 1:** Parameters of the kinetic model of the adsorbents.

Model	Parameters	QCL	PCL
pseudo-first-order kinetics	*k*_1_(h^−1^)	0.3809	0.3833
	*q_e_* (mg/g)	19.4410	20.5938
	R^2^	0.7772	0.7207
pseudo-second-order kinetics	*k*_2_ (g/(mg·min))	0.0229	0.0153
	*q_e_* (mg/g)	21.8723	23.3046
	R^2^	0.9982	0.9991
	h_0_	10.955	8.3095
Weber and Morris	*K_p_*_1_	5.0313	2.9340
	*C*_1_	3.9268	8.5383
	R_2_	0.9999	0.9905
	*K_p_*_2_	1.1318	0.6968
	*C*_2_	13.9619	16.908
	R^2^	0.9822	0.9191
Elovich model	*β* (g/mg)	0.3448	0.3780
	*α* (mg/g·min)	93.8163	177.9421
	R^2^	0.9766	0.9625

**Table 2:** Langmuir and Freundlich isotherm parameters and correlation coefficients for the adsorption of HA.

	Langmuir			Freundlich			Temkin		
Adsorbents	Q_m_ (mg/g)	*K_L_* (L/mg)	R^2^	*K_F_* (mg/g)	*n*	R^2^	*B_T_*	*K_T_*	R^2^
QCL	250.0	0.0084	0.9910	14.22	2.347	0.9914	45.07	0.1353	0.9111
PCL	333.3	0.0042	0.9827	9.331	1.939	0.9832	69.24	0.0468	0.9212

**Table 3:** Adsorption capacity of other adsorbents for HA removal.

Adsorbents	Q_m_ (mg/g)	pH	Temperature	Ref.
Fe_3_O_4_-chitosan hybrid nano-particles	44.84	4	RT	[20]
Zeolitic Imidazole Framework-8 (ZIF-8)	45	3	20 °C	[21]
Activated carbon	45.4	--	--	[22]
bentonite	53	5	25 °C	[23]
Natural Algerian bentonite	54.8	3.2	20 °C	[2]
cross-linked carboxymethyl chitosan	57.14	3.5	RT	[6]
chitosan-encapsulated activated carbon	84	6.4	30 °C	[24]
Modified HDTMA-bentonite	104.77	3.0	30 °C	[25]
ZnO-30N-zeolite	120	--	21 °C	[26]
QCL	250.0	6	30 °C	This paper
Modified HDTMA-bentonite	330.3	3.2	20 °C	[2]
PCL	333.3	6	30 °C	This paper

**Table 4:** Desorption efficiencies of adsorbed HA on PCL and QCL.

Elution Reagent	Desorption Efficiency (%)		
	PCL		QCL
1 mol/L HCl	3.268		33.12
3 mol/L HCl	7.631		35.98
1 mol/L NaOH	55.04		37.14
3 mol/L NaOH	75.98		30.86
